# Delivering an Optimised Behavioural Intervention (OBI) to people with low back pain with high psychological risk; results and lessons learnt from a feasibility randomised controlled trial of Contextual Cognitive Behavioural Therapy (CCBT) vs. Physiotherapy

**DOI:** 10.1186/s12891-015-0594-2

**Published:** 2015-06-16

**Authors:** Tamar Pincus, Shamaila Anwar, Lance M. McCracken, Alison McGregor, Liz Graham, Michelle Collinson, John McBeth, Paul Watson, Stephen Morley, Juliet Henderson, Amanda J. Farrin, on behalf of the OBI Trial Management Team

**Affiliations:** Department of Psychology, University of London, Royal Holloway, Egham, TW20 0EX Surrey, UK; Clinical Trials Research Unit, Leeds Institute of Clinical Trials Research, University of Leeds, LS2 9JT Leeds, UK; Health Psychology Section, Psychology Department, King’s College, 5th Floor Bermondsey Wing, Guy’s Campus, SE1 9RT London, UK; Department of Surgery and Cancer, Faculty of Medicine, Imperial College London, Charing Cross Hospital, W6 8RF London, UK; Arthritis Research UK Primary Care Centre, Primary Care Sciences, Keele University, ST5 5BG Keele, Staffordshire UK; Department of Health Sciences, University of Leicester, LE5 4PW Leicester, UK; Leeds Institute of Health Sciences, University of Leeds, 101 Clarendon Road, LS2 9LJ Leeds, UK

**Keywords:** Feasibility trial, Randomised controlled trial, Chronic back pain, Fear avoidance, Contexual cognitive behavioural therapy, Acceptance and commitment, Sub-groups, Physiotherapy, Acceptability & credibility

## Abstract

**Background:**

Low Back Pain (LBP) remains a common and costly problem. Psychological obstacles to recovery have been identified, but psychological and behavioural interventions have produced only moderate improvements. Reviews of trials have suggested that the interventions lack clear theoretical basis, are often compromised by low dose, lack of fidelity, and delivery by non-experts. In addition, interventions do not directly target known risk mechanisms. We identified a theory driven intervention (Contexual Cognitive Behavioural Therapy, CCBT) that directly targets an evidence-based risk mechanism (avoidance and ensured dose and delivery were optimised. This feasibility study was designed to test the credibility and acceptability of optimised CCBT against physiotherapy for avoidant LBP patients, and to test recruitment, delivery of the intervention and response rates prior to moving to a full definitive trial.

**Methods:**

A randomised controlled feasibility trial with patients randomised to receive CCBT or physiotherapy. CCBT was delivered by trained supervised psychologists on a one to one basis and comprised up to 8 one-hour sessions. Physiotherapy comprised back to fitness group exercises with at least 60 % of content exercise-based. Patients were eligible to take part if they had back pain for more than 3 months, and scored above a threshold indicating fear avoidance, catastrophic beliefs and distress.

**Results:**

89 patients were recruited. Uptake rates were above those predicted. Scores for credibility and acceptability of the interventions met the set criteria. Response rates at three and six months fell short of the 75 % target. Problems associated with poor response rates were identified and successfully resolved, rates increased to 77 % at 3 months, and 68 % at 6 months. Independent ratings of treatment sessions indicated that CCBT was delivered to fidelity. Numbers were too small for formal analysis. Although average scores for acceptance were higher in the CCBT group than in the group attending physiotherapy (increase of 7.9 versus 5.1) and change in disability and pain from baseline to 6 months were greater in the CCBT group than in the physiotherapy group, these findings should be interpreted with caution.

**Conclusions:**

CCBT is a credible and acceptable intervention for LBP patients who exhibit psychological obstacles to recovery.

**Trial registration:**

ISRCTN43733490, registered 15/12/2010.

**Electronic supplementary material:**

The online version of this article (doi:10.1186/s12891-015-0594-2) contains supplementary material, which is available to authorized users.

## Background

Low back pain continues to be a major cause of disability worldwide [1]. Psychological obstacles for recovery have been identified [2], and psychological interventions, primarily based on cognitive-behavioural approaches (CBT), have been tested in many trials. There are several systematic reviews of such trials in musculoskeletal pain populations, and in LBP specifically [3–6]. The findings across reviews suggest that CBT is marginally superior to treatment as usual, but the effect sizes for key outcomes are small (around 0.2) and the clinical significance of these effects remain unclear [7].

Several explanations have been suggested for the disappointing findings. These include diluted interventions, in which psychology is offered by non-psychologists who are untrained or trained only for the purposes of the trial; psychology offered at minimal doses; the inclusion of heterogeneous groups and the failure to select and target sub-groups who would benefit from psychological interventions; and the failure to apply theoretically-driven interventions that directly address mechanisms or processes theoretically identified and demonstrated as necessary in producing positive outcomes [8, 9].

Fear avoidance, catastrophic thinking and emotional distress have all been identified as factors contributing to poor outcomes [10, 11] indicating sub-groups with high risk. The principle of stratified care, in which patients are screened to identify sub-groups, and referred to matching interventions, has been shown to improve outcomes [12]. However the interventions, based on CBT approaches, have been less effective for high-risk groups with complex psychological factors [12]. Other psychological interventions, developed specifically to address these risk factors include exposure-based interventions. While case studies of exposure based methods have appeared promising, RCTs including these methods have appeared more limited in their success [13, 14]. The model underlying these treatments has been criticised as missing key therapeutic elements, such as specifically identified therapeutic processes, and an approach to positive goals and motivation [15]. Exposing patients to feared movements does not appear to generalise to everyday activities [16]. Optimal treatment for these patients remained unknown.

A model that may be more adequate for addressing fear avoidance, catastrophic thinking, and other emotional factors, and that includes specific therapeutic processes and an approach to goals and motivation, is the psychological flexibility model [17, 18]. Treatment approaches based on this model include Acceptance and Commitment Therapy [19] and can be more generically referred to as Contextual Cognitive-Behavioural Therapy (CCBT) [9, 20, 21].

The current study was designed to overcome the limitations associated with previous trials, by selecting patients for evidence-based risk profile and offering a theoretically-based intervention, previously untested in LBP in a full RCT, which explicitly aims to target this risk profile. The intervention was designed to full integrity, delivered by experienced psychologists extensively trained and supervised, on a one to one basis and to sufficient dose [8]. Credible control conditions have also been highlighted as an area for improvement in trials [7]. The current study included a control arm of best practice as recommended by guidelines, by offering physiotherapy [22].

The primary objectives of the study were to test the credibility and acceptability of offering CCBT to patients with high fear avoidance who had been referred to physiotherapy, assess recruitment processes and study uptake, assess the burden of measurement tool completion at baseline and follow-up time points, and test whether psychologists inexperienced in CCBT could be trained to deliver CCBT to integrity. The secondary objectives were to measure changes in Quality of Life outcomes relating to mood, pain, disability and functioning.

## Methods

### Design

We conducted a feasibility RCT comparing CCBT with physiotherapy in four NHS Musculoskeletal / Physiotherapy Services in England between September 2010 and December 2013 (see [9] for details). The study was approved by The National Health Service ethics committee, and at university level at Royal Holloway, University of London.

### Eligibility criteria

Participants experiencing chronic low back pain, suitable for physiotherapy-led treatment, classified as “avoidant” (defined via meeting set thresholds by endorsing at least one of the items measuring catastrophic beliefs, depression and fear avoidance on the Targeted Treatment for Back Pain (STarT Back) Screening Tool and scoring above 38 on the 17 item Tampa Scale for Kinesiophobia (TSK) [23]) were eligible for recruitment. The recruitment process was as follows: all referrals made to the musculoskeletal / physiotherapy services involved in the study were triaged by physiotherapy researchers. Those patients suitable were sent a screening questionnaire, and replies included consent to be contacted by the researcher. The Researcher contacted patients to further clarify eligibility and if eligible, invited the patient to attend a Physiotherapy Assessment and a face-to-face meeting with the Researcher. The Physiotherapy Assessment excluded sciatica or any other progressive disorders that may require a different clinical pathway. Eligible patients then meet face to face with the Researcher, and provided written informed consent. Baseline data were collected prior to randomization.

### Randomisation and allocation concealment

Consenting participants were randomised on a 1:1 basis to receive either CCBT or physiotherapy. This was carried out by remote computerised randomisation, which the researcher communicated to patients during the interview, after obtaining full informed consent.

### Trial interventions

1. CCBT was delivered by trial-specific psychologists trained in the intervention and supervised by the co-applicant expert (LM). Participants allocated CCBT were offered up to 8 individual sessions, of 50 min each. Session content was not structured, and at the discretion of therapists, included any features of CCBT they thought were appropriate at the point with that patient. The first session is dedicated to building a good relationship with patients and setting their expectations about the content and rationale of subsequent treatment. Subsequent sessions included a mixture of techniques based on enhancing acceptance through experiential, exposure-based, and mindfulness-based methods, using a present-moment focus, directed awareness, and values-based action. The number of sessions required by each individual patient was agreed between the patient and therapist.

Physiotherapy was delivered as usual within services, with the stipulation that it included at least 60 % exercise and comprised group sessions (in total not exceeding 8 sessions), with an allowance of up to 3 individual sessions at the start of treatment if required. This was a pragmatic trial, and physiotherapy reflected the variability within the service. The general goal of physiotherapy was to encourage re-activation. We monitored the content through self-report by the physiotherapists in short forms and observation sessions by a physiotherapist expert (AM) to ensure fidelity. However the exact content of the physiotherapy sessions could vary between therapists and patients, as is typical in the real world.

Both interventions were monitored for fidelity of treatment content and delivery.

### Data collection

Data were collected from participants at baseline via researcher interview, and via 3 and 6 months postal and telephone questionnaires post-randomisation. The following questionnaires were completed at all time-points unless otherwise stated: TSK [23], Brief Pain Inventory (BPI [24–26]), Chronic Pain Acceptance Questionnaire (CPAQ [27]), Acceptance and Action Questionnaire (AAQ-II [28]), Roland Morris Disability Questionnaire (RMDQ [29]), Short Form 12 (SF12 [30]), Hospital Anxiety and Depression Scale (HADS [31]), EuroQol-5D (EQ-5D^™^[32]), Modified Patient Global Impression of Change (PGIC [33, 34]), expectations of and satisfaction with treatment was measured using questions adapted from those used by Borkovec and Nau [35] (at baseline and 3 months). The information that all patients received at the stage of providing credibility ratings about the CCBT intervention was as follows:

- On the information sheet: If you are having the behavioural treatment, CCBT. “*Behavioural” means the treatment is aimed at training you in skills and in changing behaviour patterns to improve your health and daily activities. Both treatments are known to be safe and are already used in some hospitals.* you will need to come for a meeting with a psychologist (who specialises in helping people in pain) once a week for up to 8 weeks.
- Assessing physiotherapist: ‘There are several interventions for your type of pain, and currently it is not known which of them is most effective. Two of them are physiotherapy and a talking therapy called Contextual Cognitive Behavioural Therapy- CCBT.’
- Pre randomisation, by the researcher consenting participants: ‘CCBT involves a psychologist talking to patients about their back pain including their thoughts and feelings about pain. This is to help them understand how to make changes to their thoughts and behaviour and to lead a fuller life.’

### Qualitative study

To qualitatively assess acceptability and credibility of treatment, recruitment and follow-up processes, participants were interviewed at 3 months and therapists were interviewed at the end of the study.

### Sample size

Being a feasibility study, formal power calculations were not appropriate as the study was not designed to test for a difference between treatments. We followed recommendations to have a minimum of 30 participants per group [36], and set a target of 92 recruited participants was set, allowing for 35 % loss of data (25 % due to loss to follow-up and 10 % due to non-compliance).

The study was approved by the West London Research Ethics Committee (Reference: 11/H0706/9) and funded by Arthritis Research UK (Grant reference: 19401).

### Analyses

Combinations of qualitative and quantitative measures were used to address the primary research questions of assessing the acceptability and credibility of CCBT to patients and therapists; the viability of recruitment processes and acceptability of outcome measures for a future definitive trial. Analyses conducted were mainly descriptive and focussed on confidence interval estimation, rather than hypothesis testing, to provide estimates of the key trial parameters to determine whether to proceed to a larger definitive RCT. All analyses were conducted on an intention-to-treat basis with participants being analysed according to their randomisation allocation. Data were analysed at the end of the study when all data collection, entry and validation was completed. A-priori criteria to proceed to a larger definitive trial were established upfront in consultation with the Trial Steering Committee and are detailed below.

Credibility and acceptability scores were summarised by group. To assess credibility, mean scores and 95 % confidence intervals were calculated overall and by randomised group for the first two questions of the Borkovec and Nau expectation and satisfaction questionnaire [35] (details in Table 2). To assess acceptability, mean scores and 95 % confidence intervals of each of the five Borkovec and Nau questions were calculated by randomised group. ANCOVA (adjusting for baseline value as a covariate) was also used to calculate the change from baseline to three month scores for the first two Borkovec and Nau, together with 95 % confidence intervals. In addition, treatment dropouts, the number of participants missing treatment sessions and withdrawing (from treatment, follow-up or both) in each arm was summarised.

To further assess acceptability, qualitative analyses were conducted independently by two researchers on the participant three-month interviews (TP & JH). Directed content was used to explore consensus, and present the analysis backed by verbatim quotes to illustrate main points. Errors, omissions and commission were discussed and resolved between the researchers. Information from interviews with practitioners at the end of the trial was discussed and synthesised with data from patients, where appropriate.

Recruitment and follow-up strategies were measured by summarising eligibility, consent and randomisation rates both overall and by centre. The acceptability of measurement tool completion was assessed by summarising follow-up response rates overall and by arm at all time-points, by assessing the level of missing data (for individual items and for entire outcome measures) and via responses provided during the three-month participant interview.

### Proof of principle and secondary analyses

Proof of principle was measured by change from baseline to three and six months on CPAQ Total score, summarised by point estimated and 95 % confidence intervals. Secondary outcome measures relating to QOL (BPI, TSK, RDQ, SF12, Hospital Anxiety and Depression Scale) and recovery post treatment at six months follow up were summarised by point estimates and 95 % confidence intervals and presented by randomised group, at each time point.

### A-priori criteria for credibility, acceptability and feasibility

The following *a-priori* criteria were established to assess whether to proceed to a larger definitive trial.

Criteria for acceptability and credibility were set at a score of at least five points out of the maximum of 10 on each of the Borkovec and Nau items [9]. The mid-point response was considered sufficient in consideration that patients would have been referred to physiotherapy, and were expected to therefore have higher expectations for physiotherapy.

An acceptable level of treatment completion was set at 90 % of randomised patients completing allocated treatment. This definition does not take into account the number of sessions attended, because individual patients may have required different doses of treatment. Completion was therefore defined as cases where both therapist and patient agreed that treatment was completed.

To proceed to a larger trial, at least 3 % of all those referred should be recruited and at least 20 % of patients completing a screening questionnaire should be recruited. In addition loss of data for analysis should not exceed 35 % post randomisation (10 % due to non-compliance and 25 % loss to follow up).

To demonstrate proof of principle, the improvement in CPAQ and the AAQ-II scores should be greater in the group receiving CCBT than the control group, but this could not be tested explicitly due to the lack of power in our small sample [37]. Instead we planned to report means and 95 % CI.

## Results and discussion

### Recruitment

**Fig. 1:**
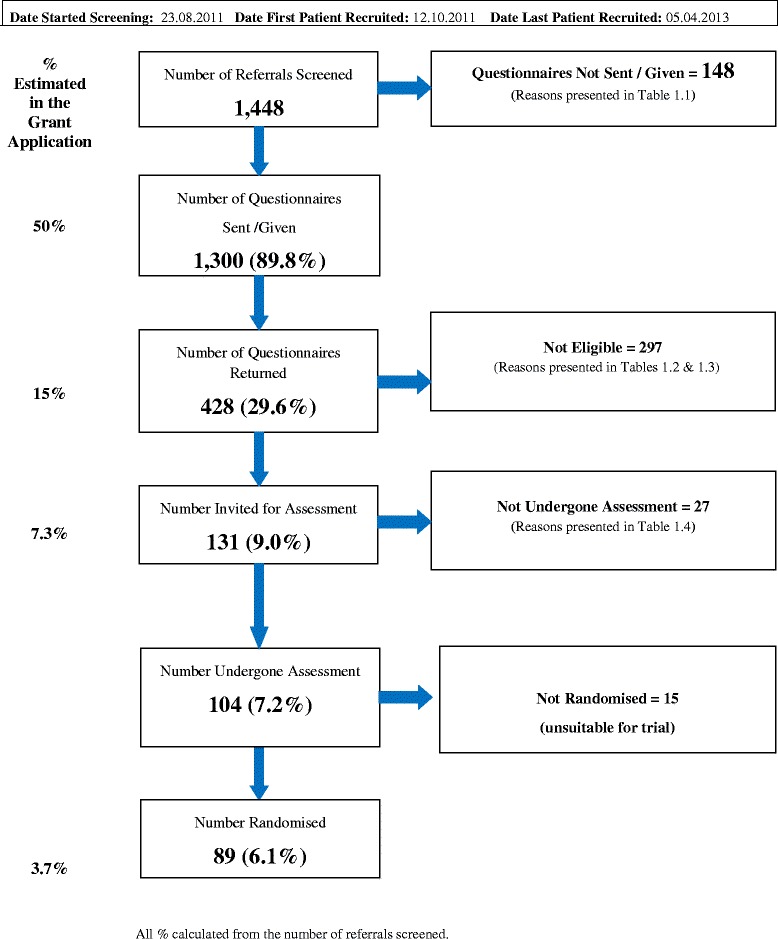
Flow chart of recruitment (Additional file 1)

The recruitment process is described in Fig. 1. Between August 2012 and April 2013 1448 referrals were screened from four centres across England of which 1,300 (89.8 %) were sent screening questionnaires. 131 (9.0 %) of those referred and sent questionnaires appeared eligible upon receipt of completed questionnaires. After physiotherapy assessment for eligibility (n = 104), 89 participants were randomised into the trial (45 in the CCBT group and 44 in the Physiotherapy group), comprising 86 % of those eligible to attend for physiotherapy assessment (Fig. 1). Recruitment between centres differed: two centres relied on referrals from pain services and pain consultants, and did not recruit sufficient numbers into the trial (n < 5), so recruitment was suspended in these two centres. Exploration of the reasons for not recruiting suggested lack of equipoise and insufficient number of lower back pain referrals without further complications. The remaining two centres were large primary care-based Musculoskeletal / Physiotherapy Services with referrals coming from GP practices.

### Participants’ characteristics

The intervention groups were well balanced at baseline for most characteristics (Table 1). There were more female participants than male participants in both groups. BPI pain interference score was similar between the groups. The CCBT group had a shorter duration of pain than the Physiotherapy group (median of 27 vs. 36 months). 44 % of participants indicated that this was their first episode of pain, but all had experienced pain for more than three months. When compared to the CCBT group, around 20 % more participants in the Physiotherapy group were working full / part-time at baseline. The majority of those recruited had received formal education, and just over half of these left school aged 16 or less.

**Table 1:** Baseline characteristics by arm

	CCBT (n = 45) N (%)	Physiotherapy (n = 44) N (%)	Total (n = 89) N (%)
Gender: Male	18 (40.0 %)	17 (38.6 %)	35 (39.3 %)
Age (years): Mean (standard deviation)	43.7 (16.33)	45.4 (15.82)	44.6 (16.01)
Main employment status			
Working full time	18 (40.0 %)	24 (54.5 %)	42 (47.2 %)
Working part time	5 (11.1 %)	7 (15.9 %)	12 (13.5 %)
At home and not looking for work	5 (11.1 %)	2 (4.5 %)	7 (7.9 %)
Unemployed and looking for work	7 (15.6 %)	1 (2.3 %)	8 (9.0 %)
Retired	8 (17.8 %)	7 (15.9 %)	15 (16.9 %)
Unable to work	1 (2.2 %)	2 (4.5 %)	3 (3.4 %)
Other	1 (2.2 %)	1 (2.3 %)	2 (2.2 %)
Age participant left full time education	(n = 43)	(n = 41)	(n = 84)
16 or less	25 (58.1 %)	21 (51.2 %)	46 (54.8 %)
17-20	12 (27.9 %)	13 (31.7 %)	25 (29.8 %)
21 or over	6 (14.0 %)	6 (14.6 %)	12 (14.3 %)
Unknown	0 (0.0 %)	1 (2.4 %)	1 (1.2 %)
BPI pain interference score < =5	25 (55.6 %)	25 (56.8 %)	50 (56.2 %)
Duration of current episode of lower back pain (months)	(n = 45)	(n = 43)	(n = 88)
Mean (standard deviation)	42.4 (37.97)	44.6 (36.09)	43.5 (36.87)
Median (range)	27.0 (3.0, 99.0)	36.0 (3.0, 99.0)	36.0 (3.0, 99.0)
First episode of pain: Yes	21 (46.7 %)	18 (40.9 %)	39 (43.8 %)
Other treatment received in the past	(n = 24)	(n = 26)	(n = 50)
Physiotherapy	12 (50.0 %)	19 (73.1 %)	31 (62.0 %)
Medication	17 (70.8 %)	18 (69.2 %)	35 (70.0 %)
Manipulation	8 (33.3 %)	7 (26.9 %)	15 (30.0 %)
Acupuncture	5 (20.8 %)	4 (15.4 %)	9 (18.0 %)
Chiropractor	1 (4.2 %)	1 (3.8 %)	2 (4.0 %)
Osteopath	2 (8.3 %)	0 (0.0 %)	2 (4.0 %)
Other	3 (12.5 %)	6 (23.1 %)	9 (18.0 %)
None	3 (12.5 %)	2 (7.7 %)	5 (10.0 %)

### Training and supervision frequency

The number of training sessions varied between psychologists, but all received four full days of face-to-face training and were given reading to complete in their own time. The pattern of supervision was similar across therapists, with a weekly hour telephone session required in the first month or two, as part of training, followed by one every two weeks for another two months, and finally a single monthly session.

### Acceptability and credibility

Patients randomised to CCBT indicated that the intervention was credible (as average scores for the first two questions on the Borkovec and Nau Expectation and Satisfaction Questionnaire [35] exceeded the set cut-point of 5, Table 2). Nonetheless, and as expected, scores were higher in the group randomised to the original referred treatment, physiotherapy (mean of 6.7 vs. 8.9, and 6.6 vs. 8.1 respectively). Mean scores in the CCBT arm at three months for four of the Borkovec and Nau questions were above 5, but one item (How successful do you think this treatment will be / was in reducing the impact of pain?) was rated 4. For physiotherapy, credibility scores ranged between 6.2 and 7.4. When adjusted per protocol for baseline scores, the first two questions (which ask how logical the treatment seemed, and about treatment’s success in reducing the impact of pain) were 5.4 (95 % CI 4.43, 6.46) and 4.2 (95 % CI 3.10, 5.39) for CCBT and 6.5 (95 % CI 5.47, 7.51) and 6.0 (95 % CI 4.91, 7.05) for physiotherapy.

**Table 2:** Average raw Borkovec & Nau scores by arm and by time point

	Baseline						3 months					
Question	CCBT Mean (SD)	N	Physiotherapy Mean (SD)	N	Total Mean (SD)	N	CCBT Mean (SD)	N	Physiotherapy Mean (SD)	N	Total Mean (SD)	N
Q1 – How logical does / did the treatment offered seem to you?	6.7 (2.89)	44	8.9 (1.62)	44	7.8 (2.58)	88	5.0 (3.28)	31	6.9 (2.70)	31	6.0 (3.13)	62
Q2 – How successful do you think this treatment will be / was in reducing the impact of pain?	6.6 (2.69)	44	8.1 (1.73)	44	7.3 (2.38)	88	4.0 (3.26)	27	6.2 (2.73)	31	5.2 (3.17)	58
Q3 – How confident would you be in recommending this treatment to a friend?	N/A		N/A		N/A		5.4 (3.82)	27	7.4 (2.86)	31	6.5 (3.47)	58
Q4 – How interesting and engaging was the treatment overall?	N/A		N/A		N/A		6.5 (3.04)	27	6.4 (3.07)	31	6.4 (3.03)	58
Q5 – How satisfied were you with the overall quality of the treatment?	N/A		N/A		N/A		6.6 (3.10)	27	7.0 (3.04)	31	6.8 (3.05)	58

Only the first two questions of the Borkovec & Nau were asked at baseline. The baseline questionnaire was completed post randomisation but prior to starting treatment

Neither group required on average as many sessions as anticipated. Attendance at treatment was similar between the groups, participants in the CCBT group attended on average 2.6 sessions (2.6 SD), whilst those in the Physiotherapy group attended on average 2.3 sessions (2.3 SD).

Interviews with the CCBT group indicated that patients attending CCBT had a strong preference for one-to-one therapy. Just under half of those interviewed stated a preference for receiving some physiotherapy alongside CCBT. Patients also reported that strategies learnt during CCBT could be recalled, and used outside of treatment, and that outcome measures were clear and of acceptable length.

Interviews with therapists suggested that the while the training plans worked, there was a need to apply the acquired treatment skills with patients and complete practice in delivery with at least three patients before therapists felt competent to offer CCBT to a standard they regarded as competent. Therapists also reported that at times they felt introducing the concept of CCBT to patients was more difficult because patients knew they had been referred to physiotherapy, and therefore believed they required physiotherapy. Therapists would have preferred patients to receive some physiotherapy alongside CCBT. Finally, therapists reported that despite the screening criteria that had been designed to select patients with high avoidance levels and psychosocial obstacles to recovery, they felt that some patients were reasonably adjusted and coping, and needed very little CCBT.

### Feasibility

Overall follow-up rates were 73.0 % at three months (33 CCBT participants; 32 Physiotherapy participants) and 60.7 % at six months (26 CCBT participants; 28 Physiotherapy participants). From June 2012, around half way through the study, procedures for collecting outcome data were enhanced (additional reminders, option to complete with the researcher, and introduction of incentives), to address low response rates. Response rates were higher at both three and six months after these processes were implemented (76.9 % vs. 62.5 % at three months and 67.5 % versus 16.7 % at six months). Analysis of the three-month interviews suggested that patients felt the measures they were asked to complete were clear, and the length of the questionnaire was acceptable.

### Fidelity

Independent review of a sample (n = 25) of CCBT session audiotapes, using a structured coding format developed by LM indicated that an acceptable level of CCBT had been delivered. These were sampled to include all therapists, and instances of first, middle and last sessions. The reviewer reported that the majority of recorded sessions showed evidence for the key CCBT processes being addressed including Cognitive Defusion, Acceptance, Present Moment Awareness and Values-based Action. Therapists particularly frequently addressed ‘Exploration of feelings/ sensations’ in depth and less often demonstrated observable behaviours addressing ‘Self as Context, which might indicate a training need’. The reviewer concluded that CCBT was delivered to fidelity and that there was very little evidence of the use of traditional CBT methods.

Physiotherapy fidelity was established through a) exit interviews with a sample of patients, b) observation of one session in each centre by an expert physiotherapist from the research team, and c) through exploration of the physiotherapy session rating forms, which detailed the components covered in each session.

### Proof of principle and secondary outcomes

The majority of outcomes of both groups improved, on average, over six months post randomisation (Table 3). Changes from baseline to six months in acceptance scores suggested that CCBT increased acceptance more than did physiotherapy with an increase, on average of 7.9 on total acceptance (CPAQ) in CCBT compared to 5.1 in physiotherapy. At baseline, mean scores for CCBT and physiotherapy for the RDQ were 11.6 (4.78) and 11.7 (5.27), at three months, 9.0 (6.31) and 8.9 (6.74) and at 6 months, 7.1 (4.10) for CCBT and 8.8 (5.64) for physiotherapy. No adverse events were reported.

**Table 3:** Baseline adjusted means and 95 % confidence intervals by measure, arm and time point comparison

	Baseline – 3 Months				Baseline - 6 months			
Questionnaire	CCBT Mean (95 % CI)	N	Physiotherapy Mean (95 % CI)	N	CCBT Mean (95 % CI)	N	Physiotherapy Mean (95 % CI) N	N
Brief Pain Inventory – Pain Severity Index	14.9 (12.38, 17.46)	23	15.0 (12.64, 17.42)	26	13.8 (10.39, 17.17)	23	13.7 (10.31, 17.09)	23
Brief Pain Inventory – Function Interference Index	25.6 (19.20, 31.92)	21	23.8 (18.11, 29.52)	26	21.6 (15.88, 27.24)	23	25.4 (19.56, 31.17)	22
Chronic Pain Acceptance – Activity Engagement	42.0 (38.16. 45.90)	29	44.4 (40.72, 48.07)	32	43.5 (39.93, 47.07)	25	45.5 (42.02, 49.02)	26
Chronic Pain Acceptance ^#^ – Pain Willingness	-		-		26.0 (22.79, 29.23)	25	23.3 (20.12, 26.43)	26
Chronic Pain Acceptance – Total Score	65.6 (61.43, 69.82)	29	66.8 (62.80, 70.78)	32	69.3 (64.23, 74.44)	25	69.0 (63.96, 73.96)	26
Acceptance and Action	21.7 (19.27, 24.22)	32	18.3 (15.81, 20.76)	32	20.6 (18.03, 23.13)	26	19.1 (16.56, 21.67)	26
Roland Morris Disability	9.4 (7.60, 11.17)	23	8.6 (6.94, 10.30)	26	7.3 (5.86, 8.75)	23	8.6 (7.16, 10.12)	22
Hospital Anxiety and Depression - Anxiety	7.7 (6.42, 9.07)	23	7.65 (6.45, 8.85)	27	7.8 (6.36, 8.96)	23	7.2 (5.87, 8.47)	23
Hospital Anxiety and Depression - Depression	5.3 (4.26, 6.25)	22	5.4 (4.44, 6.28)	26	4.5 (3.34, 5.65)	23	4.8 (3.61, 5.92)	23

The TSK is not included in the table above as it could not be appropriately analysed due to a significant interaction effect between treatment and score at screening

^#^ The CPAQ Pain Willingness subscale could not be appropriately analysed between 3 months and baseline due to a significant interaction effect between treatment and score at baseline

Negative differences indicate higher scores in the Physiotherapy arm whilst positive differences indicate lower scores in the Physiotherapy arm

### Discussion

This feasibility study demonstrated that CCBT was credible and acceptable to patients with LBP, and that psychologists could be trained to deliver CCBT to integrity in a reasonably short time frame.

However, findings from both patients and therapists suggest that patients randomised to CCBT should also receive a minimal amount of physiotherapy in conjunction with CCBT. In this study, credibility ratings were a little lower for CCBT than for physiotherapy as usual. A full trial that provided a combination of physiotherapy and CCBT against physiotherapy alone would probably achieve more equal ratings of credibility. In addition, despite the fact that small numbers did not enable inferential testing, changes in both acceptance and disability were greater in the group receiving CCBT than the control physiotherapy, suggesting that the intervention is promising.

### Strengths and weaknesses

This study was designed to address limitations identified with current trials of psychological intervention for people with back pain. Our intervention was theoretically matched to known risk factors, and we attempted to combat the common practice of diluting the intervention by delivery from trained and supervised clinical psychologists, in one to one sessions, and at a credible dose. Despite this the study has limitations. This was a feasibility trial, and as such, was not powered to inform on the superiority of either of the interventions. The findings suggest that there is room to improve delivery (through inclusion of physiotherapy in conjunction with CCBT); methodology, especially in reference to collecting longer-term outcome data. The study findings are based on treatment delivery of CCBT by two therapists (although we trained four), in two centres, and therefore may not represent wider populations of patients and therapists.

Both qualitative and quantitative data suggested that some patients were well adjusted on entry to the study, despite our screening thresholds. Improvements on both screening and eligibility criteria are therefore indicated for a definitive trial, to ensure that the trial recruits the patients most likely to benefit from CCBT. For example, despite using fear-avoidance (38+ on the TSK), and a single item on the psychological sub-set of the STartBack Tool as entry requirements, therapists often reported that they considered patients to be reasonably adjusted. Considering the cost implications of providing one to one CCBT sessions, a more stringent cut point for high risk may be more useful. For the sample in this study, 8 sessions were not needed, and most patients successfully completed treatment with only 2–3 sessions. This may not be sufficient for a population with more severe adjustment problems, and considering the criticism of other trials compromising psychological interventions by reduced dose, the dose offered in a definitive trial will need careful consideration.

Our findings suggest that the best placed centres to recruit to a definitive trial are centres in which there is a steady referral from general practices to physiotherapy, rather than recruitment through pain services in secondary care. In reference to lower than expected response rates, the findings suggest that this is a particular risk for a definitive trial, possibly because of the complexity of the target population. We devised strategies to increase the response rate to an acceptable level at three months and close to acceptable levels at six months, through maximising the use of reminders, phone calls and incentives.

Although we succeeded in monitoring fidelity through audiotaping in this pilot study, this method is unlikely to be practical in a large trial. Audiotaping sessions during training is probably a useful practice, but routine monitoring of delivery in a definitive trial might need to be done through form completion by therapists and analysis of exit interviews, accompanied by a small sample of audiotaped sessions.

Finally, around half of the participants in the current study reported leaving school before age 16. This might have impacted on their ability to grasp and use some of the more cognitive content of the CCBT intervention. We note, however, that the experiential nature of the CCBT approach would be more suitable than other interventions that rely more heavily on intellectual and verbal input.

### Fit with other studies

CCBT belongs within interventions that are largely based on psychological flexibility model [18], of which the most common label is Acceptance and Commitment therapy (ACT). ACT, which formed the basis for our intervention, has been tested with several other groups of patients, and continues to show promise. ACT is now recommended as a priority for research in several guidelines [36, 38]. However, a review of the evidence suggests that the need for a definitive trial in low back pain populations remains a priority: We identified 14 RCTs in other pain populations including pilot studies: 3 large (n ≥100), 5 medium (100 > n ≥50) and 6 small (n <50). Overall, reviews of trials that include ACT-related interventions [39] conclude that acceptance-based interventions appear to be beneficial in chronic pain conditions, although currently the evidence is limited. Most recommend conducting well-designed large-scale trials to ascertain the effectiveness. The information from the RCTs suggests acceptance-based interventions, such as ACT, have some benefit for chronic pain, however, there is a lack of high-quality quality evidence and further large-scale RCTs are warranted.

### Implications

These findings, together with other evidence, help direct future trials. We should now build on our knowledge that we need to a) empower patients to make sustainable lifestyle changes within a biopsychosocial framework, b) tailor our treatments to the needs of the individual, and c) combine the expertise of physical therapists and psychologists- not just a single professional discipline - working together.

This means targeting outcomes that are applicable to daily living and easily generalise across life-spheres, screening patients and matching interventions to their specific needs, and creating cross-disciplinary synergies to deliver theoretically-fused interventions with agreed goals. Specifically, our findings suggest that a definitive trial of optimised behavioural treatment for patients with LBP who are high risk could offer CCBT, but should include a synthesis of physiotherapy and CCBT delivered by highly skilled clinicians.

## Conclusion

The findings from this study suggest that CCBT is credible, acceptable and promising for people with LBP who are also experiencing high levels of avoidance and distress. There is a need for a definitive trial using CCBT or related interventions, optimised by delivery, but combined with physiotherapy, to test improvements long term against credible controls.

## Additional file

Additional file 1:
**Details to accompany Figure 1.**

## Abbreviations

AAQ-II: Acceptance and Action Questionnaire-II
BPI: Brief Pain Inventory
CCBT: Contextual Cognitive Behavioural Therapy
CPAQ: Chronic Pain Acceptance Questionnaire
EQ-5D: Euroquol 5D
HADS: Hospital Anxiety and Depression Scale
NHS: National Health Service
RCT: Randomised Controlled Trial
RDQ: Roland Morris Disability Questionnaire
SF-12: Short-Form 12
STarT BACK: Sub-groups for Targeted Treatment for Back Pain Screening
TSK: Tampa Scale for Kinesiophobia
