# Supplementary material for: Delivering an Optimised Behavioural Intervention (OBI) to people with low back pain with high psychological risk; results and lessons learnt from a feasibility randomised controlled trial of Contextual Cognitive Behavioural Therapy (CCBT) vs. Physiotherapy
Source: BMC Musculoskelet Disord. 2015 Jun 16;16:147. doi: 10.1186/s12891-015-0594-2 (PMC4468803; doi:10.1186/s12891-015-0594-2)
Supplement: Additional file 1: — Details to accompany Figure 1. [file 12891_2015_594_MOESM1_ESM.docx]

**Table 1.1 – Reasons questionnaires not sent / given**

**26** - Not suffering from chronic lower back pain

**15** - Not proficient in English

**11** - Symptoms not present for >3 months or more

**10** - Diagnosed with Sciatica

**7** - Diagnosed with a progressive disorder

**4** - Under 18 years of age

**4** - Receiving psychological treatment

**3** - Not suitable for physiotherapy-led treatment

**1** – Requires referral elsewhere

**Table 1.2 – Reasons for ineligibility**

**64** - Not fear avoidant (25 No STarT Back, 39 Low TSK)

**61** - Not suitable for physiotherapy-led treatment

**31** - Stated preference for a particular treatment

**29** - Requires referral elsewhere

**27** - Diagnosed with Sciatica

**26** - Not interested

**15** - Not proficient in English

**9** - Diagnosed with a progressive disorder

**9** - Not suffering from chronic lower back pain

**7** - Receiving psychological treatment

**5** - Symptoms not present for >3 months or more

**3** - Undergoing current litigation

**3** - Already receiving treatment

**3** - Too busy

**2** - Waiting for test results / scans

**1** - Could not attend timetables CCBT / Physio sessions

**1** - Pregnant

**1** - Could not contact in appropriate time frame

**21** - Other (see table 1.2)

**Table 1.3 – ‘Other’ reasons for ineligibility**

| Unsure about diagnosis - to have MRI soon |
| --- |
| Already had physio sessions |
| Needs individual treatment |
| Patient requests surgery for back pain |
| Interview symptoms |
| Had previous physio for back pain - now for individual sessions |
| Other problems |
| Not suitable for group |
| To have an epidural |
| Patient states unable to concentrate and not able to fill out forms, questionnaires etc |
| Needs one to one treatment |
| Leg pains not lower back pain |
| Not able to complete questionnaires |
| Changed mind due to long wait to get through |
| Needs individual care plan |
| Re-contact when back from holidays |
| Not eligible (x3) |
| Missing (x2) |

**Table 1.4 – Reasons not undergone assessment**

**1** – Changed appointment with physio and no-one told me

**1** – Wants physio due to insurance claim

**1** – Too busy

**1** – Not interested

**1** – DNA, been discharged

**22** - Other
